# Machine learning assessment of retinal blood flow links metabolic dysfunction and accelerated microvascular aging

**DOI:** 10.1038/s41598-025-32776-3

**Published:** 2026-01-07

**Authors:** Shigeyuki Magi, Takahiro Maruyama, Seiji Takagi, Atsuhiko T. Naito, Yuichi Hori

**Affiliations:** 1 Division of Cell Physiology, Department of Physiology, School of Medicine, Toho University, Omori-Nishi 5-21-16, Ota-Ku, Tokyo, Japan; 2 Department of Ophthalmology, Toho University Omori Medical Center, Omori-Nishi 6-11-1, Ota-Ku, Tokyo, Japan; 3 Department of Ophthalmology, Toho University Graduate School of Medicine, Tokyo, Japan

**Keywords:** Laser speckle flowgraphy, Machine learning, Microvascular aging, Retinal blood flow, Metabolic syndrome, Metabolic dysfunction–associated fatty liver disease, Biomarkers, Computational biology and bioinformatics, Diseases, Medical research

## Abstract

Microvascular aging impairs tissue perfusion and contributes to age-related organ dysfunction; however, direct approaches to assess microvascular aging remain limited. Here, we developed machine learning models to estimate chronological age from retinal blood flow data acquired by laser speckle flowgraphy (LSFG) in 1,008 adults undergoing health checkups. Using 18 predefined parameters and 3,253 automatically extracted time-series features, the best model for all participants achieved a mean absolute percentage error (MAPE) of 10.3% between predicted and chronological age. In addition, sex-specific models for women and men outperformed the model for all participants (MAPE 8.6% in women and 9.3% in men), allowing us to explore sex differences in microvascular aging patterns. Based on bias-corrected residuals, we defined a novel biomarker, the relative microvascular aging index (rmVAI). Higher rmVAI was significantly associated with elevated blood pressure, diabetes, metabolic syndrome, and metabolic dysfunction–associated fatty liver disease (MAFLD). Individuals classified as “model-predicted older” (rmVAI > 10%) had higher fatty liver index values (mean 38.2 vs 26.7) and an approximately twofold higher prevalence of hepatic steatosis (26% vs 12%) than those classified as “model-predicted younger” (rmVAI < − 10%). Our LSFG-based machine learning framework provides a scalable and non-invasive tool for quantifying microvascular aging from retinal blood flow and suggests that MAFLD is a potent systemic contributor to accelerated microvascular aging, supporting a putative “liver–microvascular axis” that warrants further investigation for early risk stratification in clinical practice.

**Supplementary Information:**

The online version contains supplementary material available at 10.1038/s41598-025-32776-3.

## Introduction

Aging is an inevitable process experienced by all living creatures, characterized by a progressive decline in the function of nearly every organ. Alterations in vascular function and structure associated with aging play a central role in this process^1^. Age-associated changes in the structure and function of arteries (hereafter referred to as arterial aging) are essential contributors to the development of cardiovascular and cerebrovascular diseases^2^. In addition, age-related changes in the microvasculature (hereafter referred to as microvascular aging) affect blood supply to all organs, thereby contributing to age-related organ dysfunction. Impairments in microvascular function and structure have been reported in the heart^3^, brain^4^, kidney^5^, retina^6^, and peripheral tissues^7^ and have been implicated in the pathophysiology of aging-related diseases such as ischemic heart disease, dementia, and chronic kidney disease.

Machine learning (ML) models are powerful tools for integrating massive amounts of data from diverse domains and extracting meaningful patterns that are often difficult for humans to recognize. In medicine, ML has been applied to disease prediction, personalized treatment, and the early detection of pathological changes by analyzing complex biological and clinical data^8^. ML models have also been developed to estimate arterial aging^9^. Non-invasive clinical parameters, such as blood pressure and pulse wave velocity (PWV), have been used as input variables in ML models to estimate arterial aging^10^. Although PWV and other hemodynamic parameters are reliable indicators of age-related progression in arterial stiffness, ML models utilizing these parameters primarily assess macrovascular or arterial aging. In contrast, ML models focusing on microvascular aging remain underexplored, despite their critical role in early vascular dysfunction and systemic aging-related diseases.

The eye is a unique organ in which the structure and function of retinal vasculature can be directly observed noninvasively. Previous studies have utilized images from fundus photography and optical coherence tomography (OCT) to construct ML models for predicting chronological age^11,12^ and assessing the risk of various diseases^11,13,14^. However, these imaging modalities primarily capture structural rather than functional changes. Since microvascular aging may precede overt structural abnormalities, a method capable of detecting functional alterations in the microvasculature is needed.

Laser speckle flowgraphy (LSFG) is a non-invasive method that quantifies blood flow dynamics by detecting changes—or ‘blurs’—in the speckle pattern of light waves reflected by moving erythrocytes^15^. Unlike fundus photography and OCT, LSFG has the potential to assess functional changes in blood flow before structural abnormalities become apparent. Given its potential to detect early microvascular dysfunction, LSFG represents a promising tool for evaluating microvascular aging.

**Fig. 1:**
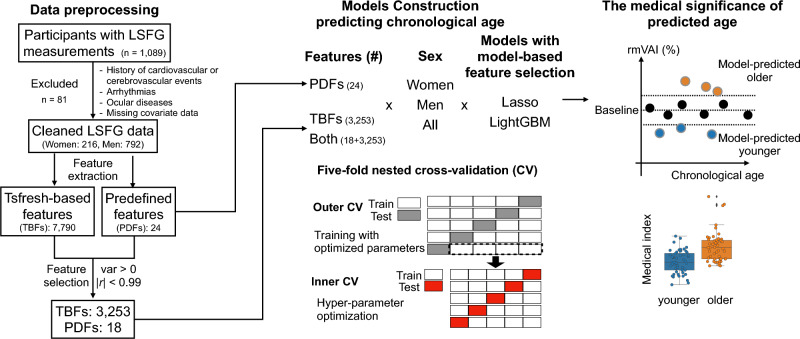
Workflow for developing machine learning (ML) models to predict chronological age from LSFG data and for subsequent analyses. A total of 1,089 adults underwent LSFG examinations during health checkups. Of these, 81 were excluded according to prespecified criteria (history of cardiovascular or cerebrovascular events, arrhythmias, ocular diseases) or due to missing covariate data, resulting in a final analytic sample of 1,008 participants (212 women and 796 men). ML models were developed using various combinations of feature sets, sex groups, and algorithms, employing five-fold nested cross-validation (CV). To assess the clinical relevance of the relative microvascular aging index (rmVAI), participants were classified into “model-predicted older” (rmVAI > 10%) and “model-predicted younger” (rmVAI < − 10%) groups. Clinical parameters were then compared between these groups to identify factors associated with accelerated microvascular aging.

In this study, we developed ML models to predict chronological age using features derived from retinal blood flow measurements obtained via LSFG in individuals undergoing routine health checkups (Fig. 1). We selected two complementary machine learning algorithms for LSFG-based age prediction: lasso regression and LightGBM. Lasso regression is a linear model with L1 regularization that performs automatic feature selection and yields a sparse, interpretable representation of high-dimensional features^16^. LightGBM is a gradient boosting decision tree method that can model non-linear relationships and higher-order interactions among features^17^. Applying both algorithms to the same LSFG feature sets and demographic groups allowed us to compare a simple, interpretable linear model with a more flexible non-linear model for estimating microvascular aging.

Using these LSFG-based age-prediction models, we then derived a relative microvascular aging index (rmVAI) from the bias-corrected residuals between model-predicted and chronological age and examined its associations with clinical parameters and metabolic risk factors. Finally, we investigated clinical and metabolic correlates of accelerated microvascular aging to explore how LSFG-based functional measures of ocular blood flow may reflect systemic vascular and metabolic risk.

## Methods

### Study design

This study was approved by the ethics committee of Toho University School of Medicine (A20037) and was conducted in accordance with the Ethical Guidelines for Medical and Biological Research Involving Human Subjects in Japan and with the principles of the Declaration of Helsinki. We used a dataset from a previous cross-sectional LSFG study of health checkup participants (A16062; UMIN Clinical Trials Registry ID: UMIN000026778). In the original study, informed consent was obtained from all participants. The present retrospective machine learning analysis was additionally registered in the UMIN Clinical Trials Registry (ID: UMIN000043408). For this secondary analysis (A20037), the ethics committee approved the use of an opt-out procedure; details of the study were disclosed on the institutional website, and potential participants were given the opportunity to decline participation. The study workflow is depicted in Fig. 1.

### Participants

From an initial pool of 1,089 individuals who underwent LSFG examinations as part of a routine medical checkup program, we excluded those with a history of cardiovascular or cerebrovascular events, cardiac arrhythmias, or ocular diseases (e.g., glaucoma, uveitis, optic neuropathy, vitreous or retinal diseases). Individuals who had undergone intraocular surgery or had missing values in the time-series mean blur rate (MBR) waveforms or key clinical covariates were also excluded. After these exclusions (n = 81), 1,008 participants (212 women and 796 men, all Japanese) were included in the analysis and model training. Because this was a health checkup cohort of generally asymptomatic middle-aged adults, the prevalence of prior cardiovascular, cerebrovascular, and advanced ocular diseases was relatively low. LSFG measurements were obtained for both eyes, when possible, but to avoid pseudo-replication and information leakage due to inter-eye correlation, only right-eye data were used for model development. Bilateral data were used in additional analyses to assess inter-eye concordance and the robustness of right-eye–based models.

### Measuring optical blood flow by using laser speckle flowgraphy

**Fig. 2:**
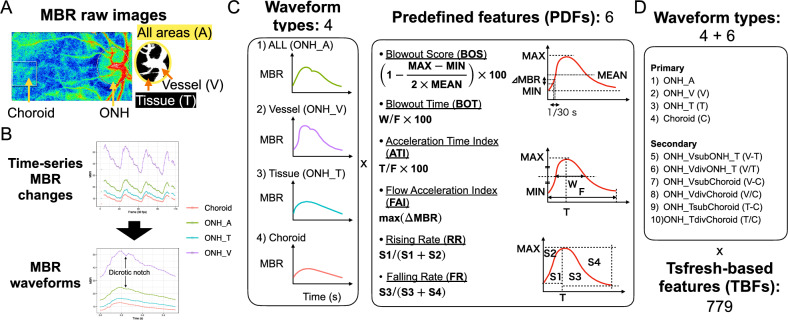
Feature extraction from LSFG images. (**A**) **Left**: Representative heatmap of the mean blur rate (MBR) captured by LSFG. An ellipse and a square indicate the optic nerve head (ONH) and the macular choroidal region temporal to the ONH, respectively. **Right**: Within the ONH, the vessel area and tissue area were analyzed separately (ONH_V and ONH_T) and also as a combined region (ONH_A). (**B**) Time-series changes in MBR from each region were normalized to generate representative MBR waveforms. A dicrotic notch can be observed in the ONH_V and ONH_A waveforms. (**C**) Six predefined features (PDFs) were calculated from each of the four MBR waveforms, yielding 24 PDFs for modeling training. (**D**) In addition to the four primary MBR waveforms, six secondary waveforms were generated by subtracting or dividing waveform pairs. A total of 779 tsfresh-based features (TBFs) were extracted per waveform, resulting in 7,790 TBFs overall.

LSFG examinations were performed as part of a routine health check-up program, and the measurement protocol has been described in detail previously^18,19^. Briefly, examinations were conducted in the morning after overnight fasting, following at least 10 min of seated rest in a quiet, air-conditioned room (24 °C), and participants were instructed to abstain from caffeine, alcohol, and smoking for at least 24 h before the visit. LSFG images were acquired with the LSFG-NAVI™ instrument (Softcare Co., Fukuoka, Japan). An ellipse and a 150-pixel square were manually positioned to delineate the optic nerve head (ONH) and the macular choroidal region temporal to the ONH, respectively (Fig. 2A). LSFG data from visible blood vessels in the ONH region, including the central retinal artery and central retinal vein, were analyzed separately from the surrounding tissue area (Fig. 2A). Time-series MBR fluctuations in the vessel (V), tissue (T), and entire ONH areas (A) (ONH_V, ONH_T, ONH_A), as well as in the choroid, were calculated from a sequence of 118 consecutive images **(**Fig. 2B, upper). A single, normalized representative MBR waveform was then generated (Fig. 2B, lower). The LSFG Analyzer software (version 3.0.47, Softcare) was used to calculate MBR and visualize MBR waveforms.

### Predefined feature (PDF) extraction from MBR waveforms

The LSFG Analyzer software automatically calculates several waveform-derived metrics, hereafter referred to as “predefined features” (PDFs)^20^. In this study, PDFs were extracted from four regions: ONH_V, ONH_T, ONH_A, and the choroid (Fig. 2C, left). Six PDFs were used: Blowout Score (BOS), Blowout Time (BOT), Acceleration Time Index (ATI), Flow Acceleration Index (FAI), Rising Rate (RR), and Falling Rate (FR) (Fig. 2C, right). All features were normalized to Z-scores, resulting in 24 PDFs (6 PDFs × 4 regions) for subsequent feature selection and model training.

### Automated tsfresh-based feature (TBF) extraction from MBR waveforms

To enhance the predictive accuracy, additional features were generated via automated feature engineering. To further expand the feature sets, six types of “secondary” MBR waveforms were created by subtracting or dividing the waveforms among ONH_V, ONH_T, ONH_A, and choroid. (Fig. 2D). The tsfresh Python package (version 1.7.0)^21^ was then used to extract a broad array of features from both primary and secondary MBR waveforms. This process generated 779 features per waveform type, yielding 7,790 tsfresh-based features (TBFs). All features were standardized to Z-scores before model training.

### Filter-based feature selection

Features with no variance or an absolute Pearson correlation coefficient (PCC) > 0.99 with any other feature were removed. PCC-based filtering was performed iteratively, starting with pairs exhibiting the highest absolute PCC. For each pair, the sum of absolute PCCs with the remaining features was calculated, and the feature with the higher sum (indicating lower independence) was excluded. During this process, two PDFs (ATI and BOS) from four MBR waveforms were found to be highly correlated with TBFs named “first_location_of_maximum” and “variation_coefficient”, respectively (Supplementary Figure S1 and S2). Among these eight pairs, seven had PCCs > 0.99, leading to the removal of six PDFs and one TBF. Finally, 18 PDFs and 3,253 TBFs were retained (Fig. 1).

### Model-based feature selection

Before nested cross-validation (CV), a “null importance” analysis^22^ was performed to select the most relevant features. This method compares the actual importance of each feature in the model with its null importance, computed using shuffled data. Initially, hyperparameters were optimized by five-fold CV with all features, using Optuna (version 2.8.0) (Fig. 1). Models were then generated to predict chronological age, and the ratio of actual-to-null importance was calculated for each feature. The optimal subset of top-ranked features that minimized prediction error was selected for final model training.

### ML models

Eighteen ML models were developed using a five-fold nested CV approach (Fig. 1), combining three feature sets (only PDFs, only TBFs, and both), three sample sets (women, men, and all participants), and two ML algorithms (lasso regression and LightGBM). In the outer CV loop, the 1,008 participants were divided into five folds, ensuring balanced sex and 12-year age strata. In each outer iteration, four folds (80% of the data) were used for model training and inner cross-validation, and the remaining fold (20%) was held out as a test set. Hyperparameter tuning was conducted in the inner CV using Optuna. Predictive performance (MAE, MAPE, and Pearson’s correlation coefficient) was evaluated on the outer test folds and averaged across the five outer iterations. For downstream analyses, we used out-of-fold predicted ages so that all residuals and rmVAI values were based on held-out predictions rather than in-sample fits. The mean absolute percentage error (MAPE) was used as the primary optimization metric to maintain predictive accuracy across the entire age range, with particular emphasis on younger participants.

### Model interpretation

Local explainability was evaluated using SHapley Additive exPlanations (SHAP)^23^, generated by the KernelExplainer in the SHAP Python package. Global explainability was assessed by averaging the local SHAP values across all participants.

### Relative microvascular aging index (rmVAI)

A linear regression analysis was conducted to correct for the systematic bias in the ML model, which tended to overestimate the ages of younger participants and underestimate the age of older participants^24–26^. First, for each participant we calculated the raw difference between the age predicted by the corresponding sex-specific model and their chronological age as:

$$Resid =(Predicted age) -(Chronological age)$$

Next, a linear regression analysis was performed using Resid as the dependent variable with chronological age and sex as independent variables:

$${\mathrm{Re}} sid \sim \, \beta^{0} + \, \beta^{1} \times (Chrono\log icalage) + \, \beta 2 \, \times (sex) + \varepsilon$$

The resulting residual ($\widehat{\varepsilon }$) for each participant represents the bias-adjusted deviation from their chronological age. This residual ($\widehat{\varepsilon }$) was then normalized by the participant’s chronological age and expressed as a percentage, defining the relative microvascular aging index (rmVAI):

$$rmVAI = \left(\frac{\widehat{\varepsilon }}{Chronological age}\right)\times 100 \left(\%\right)$$

A positive rmVAI indicates that the participant’s microvasculature appears older than their actual age, whereas a negative rmVAI suggests a younger microvascular profile. In the primary analyses, rmVAI was treated as a continuous variable. For descriptive stratification, we defined participants with an rmVAI greater than 10% as “model-predicted older” and those with an rmVAI less than − 10% as “model-predicted younger.” These thresholds were chosen to correspond approximately to one standard deviation (SD = 9.2%) of the rmVAI distribution observed in our cohort. We then evaluated the potential of rmVAI as a marker of microvascular aging by examining associations between these categories and various clinical parameters. To verify that the observed associations were not dependent on a specific threshold definition, we also examined alternative categorization schemes. Specifically, we varied the cut-offs for the “model-predicted older” and “model-predicted younger” groups to ± 8% and ± 12%, and additionally stratified participants into quartiles of rmVAI, comparing clinical characteristics between the lowest (Q1) and highest (Q4) quartiles to capture phenotypic differences at the extremes of relative microvascular aging.

### Clinical parameters

The following parameters were analyzed in relation to rmVAI and compared between the “model-predicted older” and “model-predicted younger” groups: heart rate, height, weight, body mass index (BMI), waist circumference, systolic and diastolic blood pressure (SBP, DBP), white and red blood cell counts (WBC, RBC), platelet count (Plt), hemoglobin (Hb), hematocrit (Ht), C-reactive protein (CRP), serum albumin (Alb), aspartate aminotransferase (AST), alanine aminotransferase (ALT), γ-glutamyltransferase (γGTP), creatinine (Cr), serum amylase (AMY), total cholesterol (Cho), triglycerides (TG), high-density and low-density lipoproteins (HDL, LDL), fasting plasma glucose (FPG), glycated hemoglobin (HbA1c), and current smoking status and medication use for hypertension, diabetes, and dyslipidemia. Medication use for these conditions was recorded as a binary status (current pharmacologic treatment: yes/no) based on the health checkup records. Specific drug classes, doses, and treatment duration were not systematically collected in this health-screening program and were therefore unavailable for analysis.

The fatty liver index (FLI) was originally developed to predict the presence of hepatic steatosis, with a cutoff of FLI ≥ 60 indicating steatosis. FLI was calculated using BMI, waist circumference, γGTP, and TG as follows^27^:

$$X=0.953 \times \mathrm{log}\left(TG\right)+0.139 \times BMI+0.718 \times \mathrm{log}\left(\gamma GTP\right)+0.053 \times waist circumference-15.745$$

$$FLI= \frac{{e}^{X}}{1+{e}^{X}}$$

where *X* is the logistic regression score derived from the four input variables.

The presence of metabolic syndrome (MetS) was assessed according to the Japanese criteria^28^. MetS was defined as a waist circumference of ≥ 85 cm for men or ≥ 90 cm for women, plus at least two of the following conditions: elevated fasting plasma glucose (FPG ≥ 110 mg/dL), elevated blood pressure (SBP ≥ 130 mmHg or DBP ≥ 85 mmHg), or dyslipidemia (HDL cholesterol ≤ 40 mg/dL or triglycerides ≥ 150 mg/dL).

Estimated glomerular filtration rate (eGFR) was calculated using the Japanese Society of Nephrology/CKD-EPI for Japanese Eq^29^..

### Statistical analysis

All LSFG-derived features were complete with no missing values. For clinical and laboratory variables, some had missing values in a non-negligible proportion. Missing data were handled using a complete-case approach: for each analysis, participants with missing values in any of the variables included were excluded from that analysis, and no imputation was performed. The number of participants contributing to each analysis is reported in the corresponding tables. Group comparisons of continuous variables were performed using the Mann–Whitney U test. Categorical variables were analyzed using the chi-square (χ^2^) test. To account for multiple testing in the univariable correlation analyses between rmVAI and clinical or biochemical variables, we controlled the false discovery rate (FDR) using the Benjamini–Hochberg procedure. For each correlation, both the unadjusted p-value and the FDR-adjusted q-value were calculated, and associations with q < 0.05 were considered statistically significant.

## Results

### Development of age-predicting ML models from LSFG data

**Fig. 3:**
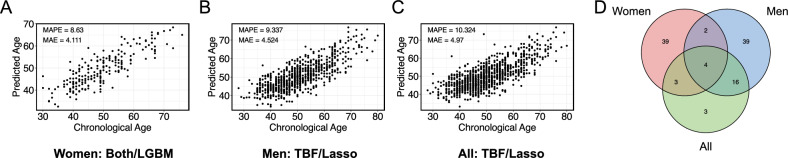
Best-performing ML models by demographic group and characteristics of selected features. (**A-C**) Scatter plots showing the relationship between actual chronological age and age predicted by the best-performing ML model for women (**A**), men (**B**) and all participants (all) (**C**). Prediction accuracy is indicated by mean absolute percentage error (MAPE) and mean absolute error (MAE). (**D**) Venn diagram showing the number of shared and unique features across the best-performing models in each demographic group.

The final study population comprised 1,008 adults (21% women), with chronological age ranging from 28 to 80 years (mean ± SD 50.1 ± 9.3 years). The age distribution was skewed toward middle age, with the largest proportion of participants in their 40 s and 50 s (Supplementary Figure S3). To develop ML models for predicting chronological age, we evaluated two algorithms: lasso regression and LightGBM, across three demographic groups (women, men, and all participants) and three feature sets. The best model under each condition was selected based on the mean absolute percentage error (MAPE), and further evaluated using the mean absolute error (MAE) (Fig. 3A-C and Table 1), and Pearson’s correlation coefficient (PCC; Supplementary Table S1).

**Table 1:** Mean absolute percentage error (MAPE) and mean absolute error (MAE) of the ML models. Models showing the best performance for women, men, and all participants are shown in bold.

Participant group	Feature set	MAPE (%)		MAE (years)	
		**Lasso**	**LGBM**	**Lasso**	**LGBM**
**Women**	PDFs	12.067	12.572	5.831	6.12
	TBFs	9.794	11.322	4.642	5.426
	Both	9.996	**8.63**	4.739	**4.111**
**Men**	PDFs	11.164	11.67	5.401	5.675
	TBFs	**9.337**	10.249	**4.524**	4.928
	Both	9.613	10.122	4.646	4.865
**All**	PDFs	11.572	12.041	5.585	5.834
	TBFs	**10.324**	10.549	**4.97**	5.049
	Both	10.329	10.654	4.968	5.117

The best model for women was a LightGBM model using both PDFs and TBFs (MAPE: 8.63%, MAE: 4.11 years, PCC: 0.838) (Fig. 3A). In contrast, In contrast, the best model for men was a lasso regression model using only TBFs (MAPE: 9.34%, MAE: 4.52 years, PCC: 0.783) (Fig. 3B). The best model for all participants was also a lasso regression model using only TBFs (MAPE: 10.32%, MAE: 4.97 years, PCC: 0.75) (Fig. 3C).

Despite using more than 200 features, the LightGBM models performed comparably to or worse than the corresponding lasso models for men and for all participants (Supplementary Table S2). Conversely, the LightGBM model outperformed lasso regression for women, despite using only 48 features (Supplementary Table S2). Across all comparisons, models using only PDFs never outperformed those incorporating TBFs (Table 1), highlighting the importance of engineered TBFs for accurate age prediction.

We next examined age-stratified performance of the sex-specific models using the feature sets that yielded the best overall performance in the main analysis (PDFs plus TBFs for women, and TBFs only for men). For each sex-specific model, we calculated the MAPE of lasso and LightGBM within three predefined age groups (< 45, 45–59, and ≥ 60 years) for women and men (Supplementary Figure S4). Lasso and LightGBM showed very similar age-related patterns, indicating no substantial algorithm-specific age trend. In contrast, we observed modest differences by sex: in men, the lowest MAPE was seen in the 45–59-year group, whereas prediction error was slightly higher in younger and older age groups. This pattern is consistent with the fact that most men in our cohort were in their 40 s and 50 s, making the training data densest in this age range.

We next examined the features used in the best-performing models for each group. The number of selected features varied across groups: 48 for women, 61 for men, and 26 for all participants (Supplementary Table S2). The overlap between models was modest; six TBFs were shared between the models for women and men, and only four features were common to all three groups (Fig. 3D).

We then investigated the waveform types (Fig. 2C) underlying the features used in model construction. The contribution of primary versus secondary waveforms was differed by group: the models for women and all participants relied predominantly on primary waveforms, whereas the model for men used both primary and secondary waveforms to a similar extent (Supplementary Figure S5A-C). Unexpectedly, features derived from non-vascular tissue areas (ONH_T and choroid) were used more frequently than those from regions containing retinal vessels (ONH_V and the entire optic nerve head, ONH_A), despite the latter including pulsatile blood flow signals from the vascular component (Supplementary Figure S5D).

We further analyzed the distributions of local SHAP values for individual features and found distinct patterns across the models (Supplementary Figure S6). Most of the top-ranked features in the model for women exhibited a bimodal distribution (Supplementary Figure S6**A**), whereas those in the models for men (Supplementary Figure S6**B**) and for all participants (Supplementary Figure S6**C**) showed predominantly unimodal distributions with greater variability. Features derived from ONH_T and the choroidal region were consistently prominent among the top features ranked by SHAP values. Specifically, these parameters accounted for 7 of the top 10 features in the model for women, 4 of 10 in the model for men, and 6 of 10 in the model for all participants.

Taken together, these results suggest that both waveform type and derived features are largely sex-specific. Moreover, they indicate that age-related changes may be more accurately captured by relatively stable microvascular blood flow in tissue regions, rather than by pulsatile fluctuations in vascular regions. This unexpected observation led us to investigate whether our LSFG-based ML models could be leveraged to define a novel index of microvascular aging.

To evaluate inter-eye consistency and the robustness of using right-eye measurements, we additionally examined a subset of participants with bilateral LSFG data (n = 903). In this subset, the 18 predefined LSFG parameters generally showed moderate-to-strong correlations between right and left eyes and applying the right-eye–trained model for all participants to left-eye data in this bilateral subset yielded an ensemble MAE of 5.51 years and a MAPE of 11.35%, only modestly worse than the performance for the right eye (Supplementary Figure S7). These findings suggest that LSFG-based estimates of microvascular aging are largely symmetric between eyes at the level of predictive accuracy, supporting the use of right-eye data for model development.

### Calculation of relative microvascular aging index (rmVAI)

**Fig. 4:**
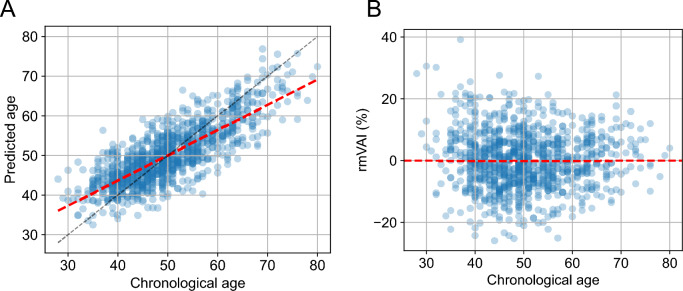
Calculation of the relative microvascular ageing index (rmVAI) and its association with clinical parameters. (**A**) Scatter plot showing the relationship between chronological age and predicted age. The gray dashed line indicates the identity line (x = y), and the red dashed line shows the regression line used to calculate the residual. (**B**) Scatter plot showing the relationship between chronological age and rmVAI. rmVAI is defined as the residual from the regression line in **(A)** divided by chronological age.

Systematic bias in ML regression models is a well-documented phenomenon across various research fields^24–26^. In our age-prediction model, we also observed that the ages of younger participants tended to be overestimated, whereas those of older participants were underestimated (Fig. 4A). To correct for this bias, we regressed the raw residuals (predicted age – chronological age) on chronological age, including sex as a covariate. The residuals from this linear model were taken as bias-corrected differences for each individual. We then normalized these bias-corrected residuals by chronological age to define the relative microvascular aging index (rmVAI). After this procedure, rmVAI was no longer correlated with chronological age (Fig. 4B), indicating that the correction effectively removed the systematic age-dependent bias of the initial model. Because rmVAI is defined as a bias-corrected residual normalized by chronological age, the same absolute prediction error corresponds to a smaller rmVAI value at higher chronological ages. Consistent with this property, the dispersion of rmVAI tended to be slightly narrower in older age groups (Fig. 4B).

### Association between rmVAI and clinical parameters

As the primary analysis, we investigated the correlation between continuous rmVAI and various clinical parameters (Table 2** and **Supplementary Table S3). A weak but significant correlation was observed between rmVAI and SBP (Pearson’s correlation coefficient [PCC] = 0.26) (Table 2), whereas other parameters showed little or no correlation (PCC < 0.20). The correlation profile between rmVAI and clinical parameters was essentially the same in men (Supplementary Table S3). In women, however, a stronger correlation was observed between rmVAI and SBP (PCC: 0.303), and SBP was the only clinical parameter that showed a statistically significant association with rmVAI.

**Table 2:** Correlations between rmVAI and clinical parameters. Pearson correlation coefficients (PCCs) between rmVAI and clinical parameters in all participants are shown. q-values represent Benjamini–Hochberg FDR–adjusted p-values for multiple testing across all clinical variables. See Supplementary Table S3 for the full set of variables and sex-specific analyses. PCCs greater than 0.20 are shown in bold. Statistical significance is indicated as follows: *q < 0.05.

Variable	Correlation	p-value	q-value
SBP (mmHg)	0.26*	1.2E-16	3.2E-15
DBP (mmHg)	0.15*	2.3E-06	3.0E-05
BMI (kg/m^2^)	0.04	2.5E-01	5.1E-01
Waist circumference (cm)	0.03	3.0E-01	5.5E-01
FPG (mg/dL)	0.11*	4.2E-04	2.7E-03
HbA1c (%)	0.12*	8.7E-04	4.5E-03
TG (mg/dL)	0.09*	5.9E-03	1.7E-02
HDL (mg/dL)	−0.02	5.5E-01	7.5E-01
LDL (mg/dL)	0.01	8.6E-01	9.6E-01
γGTP (Unit/L)	0.10*	2.2E-03	7.2E-03
WBC (10^3^/mm^3^)	0.10*	1.7E-03	6.2E-03
CRP (mg/dL)	0.05	2.1E-01	4.6E-01

We also examined whether medication influenced the correlation between rmVAI and SBP. Medication for hypertension and dyslipidemia did not affect the correlation (Supplementary Figure S8A, S8B). In contrast, medication for diabetes shifted the regression line upward (Supplementary Figure S8C) indicating that the presence of diabetes exerts an additive effect on microvascular aging.

### Clinical characterization of individuals with accelerated microvascular aging

**Fig. 5:**
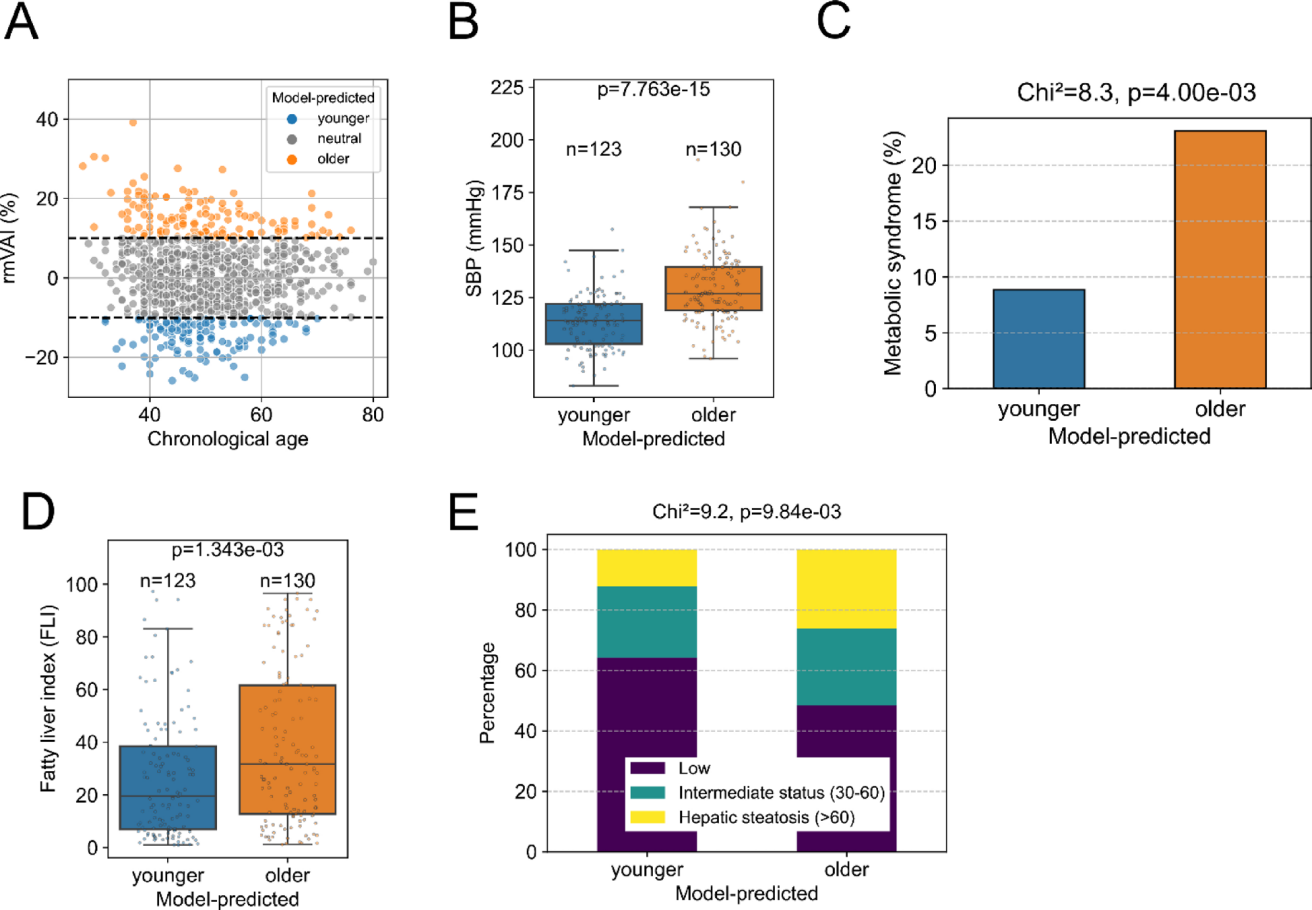
Clinical parameters associated with accelerated microvascular aging. (**A**) Scatter plot showing the relationship between chronological age and rmVAI. Colors indicate participant categories: “model-predicted younger (blue)”, “model-predicted neutral (gray)”, and “model-predicted older (orange)”. Dashed lines represent the thresholds to define the “younger” and “older” categories. (**B**) Jitter and box plot of systolic blood pressure (SBP) comparing clinical parameters between model-predicted younger and older groups. (**C**) Bar chart showing the proportion of individuals with metabolic syndrome (MetS). (**D**) Jitter and box plots of fatty liver index (FLI). (**E**) Stacked bar chart illustrating the proportion of participants classified by FLI categories. For (**B**) and (**D**), the numbers of non-missing observations and p-values from the Mann–Whitney U test are shown above each plot. For (**C**) and (**E**), Results of the Chi-square (χ^2^) test, including the χ^2^ statistic and p-value, are displayed above the plot.

To identify systemic factors potentially associated with accelerated microvascular aging, we classified individuals into groups with accelerated ("model-predicted older": rmVAI > 10%) and preserved ("model-predicted younger": rmVAI < − 10%) microvascular aging (Fig. 5A) and compared clinical parameters that differed significantly between these groups (Table 3 and Supplementary Table S3). We found that SBP and DBP were significantly higher in the accelerated microvascular aging group (130/79 mmHg vs. 114/72 mmHg) **(**Fig. 5BandTable 3). Similarly, FPG (107 mg/dL vs. 100 mg/dL) and TG (149 mg/dL vs. 112 mg/dL) were also significantly higher in the accelerated microvascular aging group, although the individual differences were modest **(**Table 3).

**Table 3:** Comparison of clinical parameters between participants predicted to be older and younger. Participants were categorized into a “model-predicted younger” group (rmVAI < − 10%) and a “model-predicted older” group (rmVAI > + 10%) based on the rmVAI. Values are shown as mean (95% confidence interval, CI) with the number of participants (n) for each group. Effect sizes are reported as mean differences (older − younger) with corresponding 95% CIs. p-values are derived from Mann–Whitney U tests, and q-values are Benjamini–Hochberg false discovery rate (FDR)–adjusted p-values calculated across all clinical parameters included in the comparison (Supplimentary Table S4).

Variable	Younger mean (95% CI), n	Older mean (95% CI), n	Mean difference (older–younger) (95% CI)	p-value	q-value
Age	48.9 (47.4–50.3), n = 124	49.3 (47.4–51.1), n = 130	0.4 (−1.9–2.7)	9.1E-01	9.1E-01
rmVAI (%)	−14.6 (−15.3–−13.9), n = 124	15.6 (14.7–16.5), n = 130	30.2 (29.1–31.3)	3.8E-43	1.0E-41
SBP (mmHg)	113.8 (111.5–116.1), n = 123	129.9 (127.1–132.8), n = 130	16.1 (12.5–19.8)	7.8E-15	1.0E-13
DBP (mmHg)	71.6 (69.9–73.4), n = 123	78.8 (76.8–80.8), n = 130	7.2 (4.5–9.8)	1.1E-06	9.9E-06
BMI (kg/m^2^)	23.2 (22.6–23.9), n = 123	24.1 (23.5–24.8), n = 130	0.9 (−0.0–1.8)	6.3E-02	1.3E-01
Waist circumference (cm)	82.8 (81.0–84.6), n = 123	85.6 (83.8–87.3), n = 130	2.8 (0.3–5.3)	1.8E-02	4.6E-02
FPG (mg/dL)	100.0 (97.3–102.7), n = 122	106.8 (103.1–110.5), n = 130	6.8 (2.2–11.3)	9.6E-03	2.8E-02
HbA1c (%)	5.6 (5.5–5.7), n = 101	5.9 (5.7–6.0), n = 97	0.2 (0.0–0.4)	1.4E-01	2.2E-01
TG (mg/dL)	111.5 (96.5–126.5), n = 123	149.3 (122.5–176.0), n = 130	37.7 (7.2–68.3)	2.6E-03	1.3E-02
HDL (mg/dL)	65.5 (62.4–68.5), n = 123	62.2 (59.2–65.2), n = 130	−3.3 (−7.5–1.0)	8.4E-02	1.5E-01
LDL (mg/dL)	125.5 (120.3–130.7), n = 123	130.5 (125.0–136.1), n = 130	5.0 (−2.6–12.6)	2.2E-01	3.0E-01
γGTP (Unit/L)	36.8 (29.2–44.3), n = 123	54.8 (42.2–67.5), n = 130	18.1 (3.4–32.7)	2.3E-03	1.3E-02
WBC (10^3^/mm^3^)	5.6 (5.3–5.8), n = 123	6.2 (5.9–6.5), n = 130	0.6 (0.2–1.0)	3.3E-03	1.3E-02
CRP (mg/dL)	0.1 (0.0–0.2), n = 99	0.2 (0.1–0.3), n = 98	0.0 (−0.1–0.2)	1.5E-01	2.3E-01

Given the modest differences in individual clinical parameters, we hypothesized that a composite indicator reflecting overall metabolic dysfunction, such as metabolic syndrome, would reveal more pronounced differences between the groups. Metabolic syndrome is a cluster of metabolic abnormalities, including central obesity, insulin resistance, hypertension and dyslipidemia, and is a known accelerator of arterial aging^30^. The prevalence of metabolic syndrome, defined by Japanese criteria, was significantly higher in the accelerated microvascular aging group compared to the preserved group (23% vs. 9%), suggesting that metabolic syndrome may contribute to the progression of microvascular aging (Fig. 5C).

### MAFLD in accelerated microvascular aging

We further investigated other systemic factors potentially associated with accelerated microvascular aging. We found that gGTP (54.8 U/L vs. 36.8 U/L) was significantly higher in the accelerated microvascular aging group (Supplementary Table S4). In contrast, AMY (69.2 U/L vs. 74.9 U/L) was significantly lower in the same group (Supplementary Table S4). Given these changes in hepatic enzymes and the previously reported association between low serum amylase level and fatty liver disease^31^, we further focused on metabolic dysfunction-associated fatty liver disease (MAFLD).

MAFLD is a chronic liver disease characterized by hepatic steatosis in individuals with metabolic dysfunction, regardless of alcohol consumption. Although limited evidence exists linking MAFLD and vascular aging, the chronic systemic inflammation associated with MAFLD may contribute to microvascular aging. The fatty liver index (FLI) is a widely used algorithm for predicting the presence of MAFLD^27,32^. FLI values were significantly higher in the model-predicted older group (38.2 vs. 26.7) (Fig. 5D). Additionally, the proportion of individuals classified as having hepatic steatosis (FLI > 60) was significantly higher in the model-predicted older group (26% vs. 12%) (Fig. 5E), suggesting that MAFLD may be an additional factor involved in accelerated microvascular aging. To assess the robustness of these findings, we re-analyzed the associations using alternative rmVAI thresholds (± 8%, ± 12%) and quartile-based stratification. We observed that the relationships between higher rmVAI and SBP, FLI, the prevalence of hepatic steatosis (FLI > 60), and MetS remained consistent in direction and magnitude across these definitions (Supplementary Figure S9).

## Discussion

In this study, we developed ML models to predict chronological age using features derived from retinal blood flow measurements obtained via LSFG. We applied these models to estimate participants’ microvascular aging and identified MAFLD and metabolic syndrome as potential pathophysiological contributors to accelerated microvascular aging. Importantly, we defined a novel index, rmVAI, based on the residuals of our ML model’s age predictions. This index serves as a distinct marker of microvascular aging, differing from conventional arterial aging indices derived from pulse-wave measures.

Investigators from various research fields have developed ML models to predict chronological age. The data used for age estimation can be broadly classified into three categories: image data (e.g., facial photographs^33^, retinal fundus images^11,12^, CT^34^, MRI^35^), which capture structural characteristics at a single time point; biochemical and physiological metrics (e.g., blood pressure, blood test results), which numerically reflect systemic health status; and functional dynamic data (e.g., LSFG, PPG, ECG, PWV), which capture real-time physiological fluctuations. Image-based models typically demonstrate high predictive precision. For example, deep learning models based on retinal images achieve a mean absolute error (MAE) of approximately 3 years^11,12^, and residuals from these models have been linked to increased mortality and cardiovascular risks. Similarly, brain MRI-based models predict age with MAEs of around 3–5 years^35^, and elevated predicted brain age correlates with neurodegenerative diseases. In contrast, ML models based on biochemical markers or physiological signals generally show slightly lower accuracy. Models utilizing blood biomarkers (e.g., albumin, glucose, urea) typically yield MAEs of around 5–6 years^36^, whereas those using functional dynamic data, such as ECG^37^ and PPG^38^, report even lower accuracy, with MAEs of approximately 6.9 and 8.1 years, respectively.

In this study, we developed ML models based on functional dynamic data obtained via LSFG. This modality captures beat-to-beat changes in retinal microvascular blood flow influenced by microvascular tone, autonomic nervous system activity, and vessel stiffness. Our models yielded MAEs of 4.1 years (women), 4.5 years (men), and 4.9 years (all participants), demonstrating superior predictive accuracy compared with previously reported dynamic functional methods such as ECG and PPG, although their performance was still lower than that of image-based models (Fig. 3A–C, Table 1). While inherent physiological variability and measurement noise associated with dynamic functional data might be considered limitations in pursuing high prediction accuracy, such variability may instead reflect subtle, early physiological changes that are not captured by static anatomical imaging or conventional biomarkers. Thus, our findings suggest that ML models using LSFG data may serve as sensitive markers for detecting early microvascular dysfunction associated with accelerated aging.

Previous studies using ML models to evaluate vascular aging have mainly focused on increased arterial stiffness associated with aging, which is an important risk factor for cardiovascular diseases. Such models typically utilize indices that reflect age-associated changes in the pulse-wave, such as PWV and PPG, to predict chronological age. The residuals of these ML-based age predictions have been proposed as markers of "accelerated arterial aging" and have been shown to correlate with cardiovascular risk^9,10,38^.

Initially, we also expected that features derived from retinal regions containing visible vessels (ONH_V), which exhibit clear pulsatile blood flow signals (Fig. 2C), would contribute more to age prediction. However, contrary to our expectation, features derived from non-vascular retinal tissue regions (ONH_T and choroid), which reflect more subtle and stable blood flow signals, were predominantly selected as critical predictors of chronological age (Supplementary Figure S5D). LSFG signals from non-vascular tissue areas primarily reflect microcirculation, including capillary blood flow that supplies tissues with oxygen and nutrients. Although less dynamic than vessel-based waveforms (Fig. 2C), microcirculation in these tissue regions is closely regulated by capillary density, microvascular tone, and autonomic nervous system activity. Our results, therefore, suggest that ML models utilizing LSFG-derived features from both vascular and non-vascular tissue areas may capture a distinct aspect of vascular aging: specifically, microvascular blood flow alterations that are not detectable through traditional pulse wave-based ML models. Such tissue-level blood flow alterations may precede or occur independently of increased arterial stiffness and macrovascular dysfunction. Impairments in tissue perfusion associated with aging are increasingly recognized as contributing factors to age-related functional decline in various organs, including cognitive deterioration in dementia^3–7^. However, we did not perform direct within-individual comparisons between LSFG-based indices and structural imaging (e.g., OCT/OCTA) or large-vessel indices (e.g., PWV/CAVI) in this cohort, and this interpretation should therefore be considered hypothesis-generating. Thus, our ML-based approach using LSFG-derived tissue perfusion features provides a novel and clinically relevant marker of microvascular aging, highlighting subtle functional abnormalities that may underlie age-related organ dysfunction.

In exploring factors associated with accelerated microvascular aging, we observed that individuals classified as "model-predicted older" exhibited a significantly higher prevalence of metabolic syndrome and MAFLD. While the association between metabolic syndrome and arterial aging is well established^30,39^, our findings linking accelerated microvascular aging to hepatic steatosis represent a novel observation. Low-grade chronic inflammation, commonly observed in MAFLD^40,41^, may play a mechanistic role in accelerating microvascular dysfunction, potentially through endothelial dysfunction, oxidative stress, or inflammatory cytokine signaling pathways. Further research is warranted to elucidate the precise mechanisms linking MAFLD-related systemic inflammation to microvascular aging, potentially uncovering new therapeutic targets for early intervention.

Several limitations of this study must be acknowledged. First, the study population was predominantly Japanese, and the generalizability of our findings to other ethnicities warrants further validation. Second, the cohort exhibited a marked sex imbalance, as approximately 80% of participants were men. Although we developed sex-specific models, this imbalance may have introduced selection bias; thus, validation in more sex-balanced cohorts is crucial to confirm the robustness of our findings, particularly in women. Third, participants were recruited from a routine health checkup program, excluding individuals with pre-existing cardiovascular or cerebrovascular events, cardiac arrhythmias, or ocular diseases. Consequently, our models were derived from a relatively healthy, middle-aged screening population, and their applicability to older age groups and to general clinical populations with multiple comorbidities remains uncertain. In addition, assessment of renal function in this setting was limited to serum creatinine and creatinine-based estimated glomerular filtration rate (eGFR), and more detailed renal phenotyping (e.g., blood urea nitrogen, albuminuria) was not available. Thus, residual confounding by subclinical kidney dysfunction, particularly in populations with more advanced renal impairment, cannot be fully excluded. However, this design aligns with our primary goal of detecting microvascular aging prior to overt clinical events in individuals who are potential targets for early risk stratification and prevention. Future studies should validate the LSFG-based models and rmVAI in hospital-based cohorts and patients with a higher comorbidity burden, across different institutions and LSFG devices.

Fourth, the cross-sectional nature of our data limits causal inference; future longitudinal studies are essential to validate the predictive accuracy and examine associations with clinical outcomes such as cardiovascular disease incidence and mortality. In addition, longitudinal follow-up and postoperative or postinterventional LSFG measurements could be used to determine whether rmVAI is a modifiable marker that tracks disease progression and therapeutic response, for example in patients with diabetes or diabetic retinopathy; such longitudinal LSFG studies are currently underway in our group. Fifth, we did not employ deep learning methods, such as recurrent neural networks or transformers, because the relatively short LSFG measurement periods limited the data volume required for training these models.

Sixth, detailed ocular parameters were not fully characterized in this study. In particular, axial length was not measured in this health-screening program, and intraocular pressure, ocular perfusion pressure, refractive error, and other ocular biometric indices were not incorporated into the present analyses. While these factors can influence ocular circulation and LSFG metrics, incorporating them would require additional specialized examinations, reducing the utility of our model as a simple screening tool. Our results suggest that LSFG-based metrics alone can sufficiently capture age-related microvascular changes for risk stratification without the need for extensive ocular biometry. Furthermore, our models relied solely on right-eye LSFG measurements. Although analyses in a bilateral subset suggested broadly symmetric performance between eyes, subtle laterality or dominance-related effects cannot be definitively excluded. Seventh, medication exposure was characterized only at a coarse level. Current treatment for hypertension, dyslipidemia, and diabetes was recorded as a binary status, whereas specific drug classes (e.g., β-blockers, calcium-channel blockers, ACE inhibitors/ARBs, diuretics, insulin, SGLT2 inhibitors, GLP-1 receptor agonists, statins), doses, and treatment duration were not available. As a result, we could not disentangle the potential effects of individual drug classes that may affect ocular blood flow, autonomic tone, or metabolism, and residual confounding by medication use in the associations between rmVAI and blood pressure or metabolic parameters cannot be excluded. Finally, although our models were developed using nested cross-validation, independent external validation is required to mitigate potential overfitting and domain shifts prior to clinical implementation.

In conclusion, our study demonstrates the potential of ML models using LSFG-derived features to identify subtle microvascular changes associated with aging. The novel relative microvascular aging index (rmVAI) may serve as a sensitive marker of accelerated microvascular aging, complementing traditional measures of arterial stiffness. This non-invasive approach could enable clinicians to identify at-risk individuals at an early stage and facilitate personalized preventive interventions. Future studies should focus on external validation, longitudinal assessment, and clinical implementation to further establish its utility in cardiovascular risk prediction.

## Supplementary Information

Supplementary Information.

## Data Availability

The datasets used and analyzed during the current study are available from the corresponding author upon reasonable request. The underlying code for this study is available on GitHub at (https://github.com/SHMAKI/LSFG_JCHO/).
