# Supplementary material for: Machine learning assessment of retinal blood flow links metabolic dysfunction and accelerated microvascular aging
Source: Sci Rep. 2026 Jan 7;16:3001. doi: 10.1038/s41598-025-32776-3 (PMC12827333; doi:10.1038/s41598-025-32776-3)
Supplement: Supplementary file 1 — Supplementary Information. [file 41598_2025_32776_MOESM1_ESM.pdf]

Title:

Machine learning assessment of retinal blood flow links metabolic dysfunction and accelerated microvascular aging

Authors:

Shigeyuki Magi Ph.D.[1†], Takahiro Maruyama M.D.[2, 3†], Seiji Takagi M.D., Ph.D.[2], Atsuhiko T Naito M.D., Ph.D.[1*], Yuichi Hori M.D., Ph.D.[2*]

1. Division of Cell Physiology, Department of Physiology, School of Medicine, Toho University, Tokyo, Japan
2. Department of Ophthalmology, Toho University Omori Medical Center, Tokyo, Japan
3. Department of Ophthalmology, Toho University Graduate School of Medicine, Tokyo, Japan

* Correspondence to: Atsuhiko T Naito, E-mail: atsuhiko.naito@med.toho-u.ac.jp, Address: Omori-nishi 5-21-16, Ota-ku, Tokyo, Japan. and Yuichi Hori, E-mail: yhori@med.toho-u.ac.jp, Address: Omori-nishi 6-11-1, Ota-ku, Tokyo, Japan.

†: equally contributed

Supplementary Figure S1. Correlation between ATI and other features

Supplementary Figure S2. Correlation between BOS and other features

Supplementary Figure S3. Age distribution of the study population in women and men

Supplementary Figure S4. Age-stratified MAPE of sex-specific LSFG-based age-prediction models

Supplementary Figure S5. Characteristics of selected features in the final models

Supplementary Figure S6. Top 10 features contributing to global explainability in the best-performing models

Supplementary Figure S7. Inter-eye consistency of LSFG-derived features and age-prediction performance in left eyes.

Supplementary Figure S8. Relationship between the relative microvascular ageing index (rmVAI) and systolic blood pressure (SBP), stratified by medication status

Supplementary Figure S9. Associations between rmVAI-defined microvascular aging status and cardiometabolic risk across alternative thresholds.

Supplementary Table S1. Pearson correlation coefficient between chronological age and predicted age in each model

Supplementary Table S2. Number of features used in the ML models.

Supplementary Table S3. Pearson correlation coefficients (PCCs) between rmVAI and clinical parameters in each sex-group.

Supplementary Table S4. Detailed summary statistics and effect sizes for clinical parameters shown in Table 4

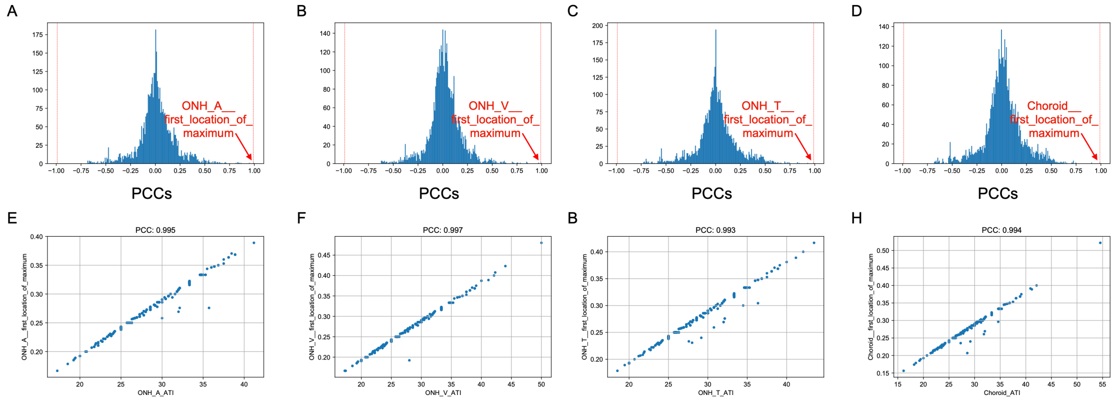

Supplementary Figure S1. Correlation between ATI and other features. **(A-D)** Histograms of Pearson correlation coefficients (PCCs) between each feature and ATI calculated from four regions: ONH_A **(A)**, ONH_V **(B)**, ONH_T **(C)**, and Choroid **(D)**. Red dashed lines indicate -0.99 and 0.99, thresholds for feature selection. **(E-H)** Scatter plots showing the relationship between ATI and the feature “first_location_of_maximum” calculated from MBR waveform in ONH_A **(E)**, ONH_V **(F)**, ONH_T **(G)**, and Choroid **(H)**. All PCCs of these pairs were over 0.99.

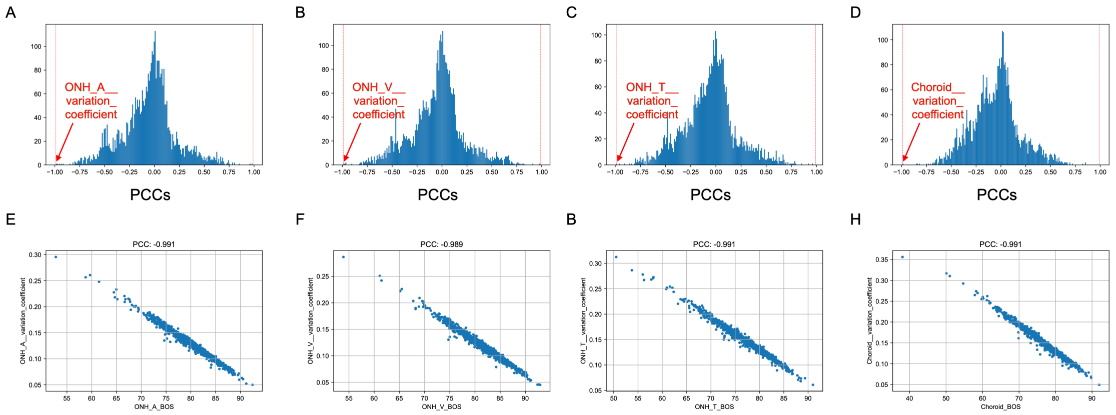

Supplementary Figure S2. Correlation between BOS and other features. **(A-D)** Histograms of Pearson correlation coefficients (PCCs) between each feature and BOS calculated from four regions: ONH_A **(A)**, ONH_V **(B)**, ONH_T **(C)**, and Choroid **(D)**. Red dashed lines indicate -0.99 and 0.99, thresholds for feature selection. **(E-H)** Scatter plots showing the relationship between BOS and the feature "variation_coefficient" calculated from the MBR waveform in ONH_A **(E)**, ONH_V **(F)**, ONH_T **(G)**, and Choroid **(H)**. All PCCs for these pairs were less than -0.99.

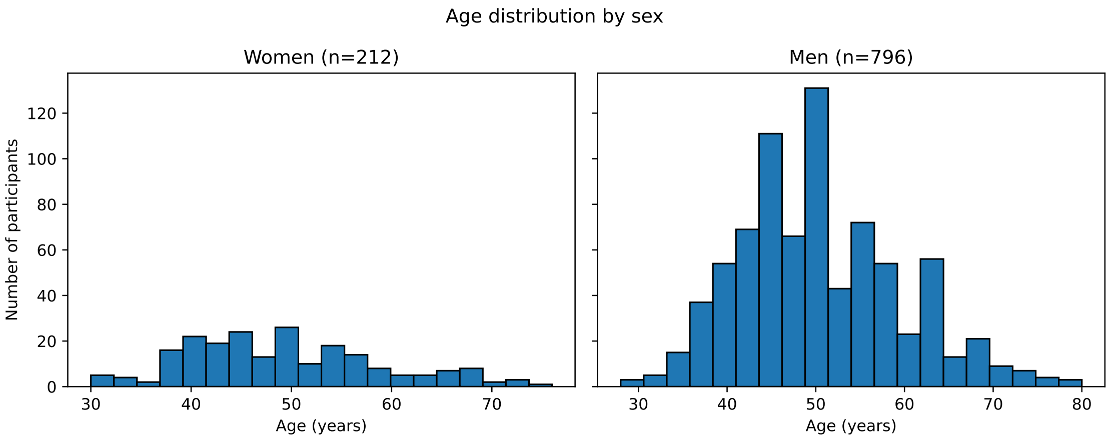

Supplementary Figure S3. Age distribution of the study population in women and men.

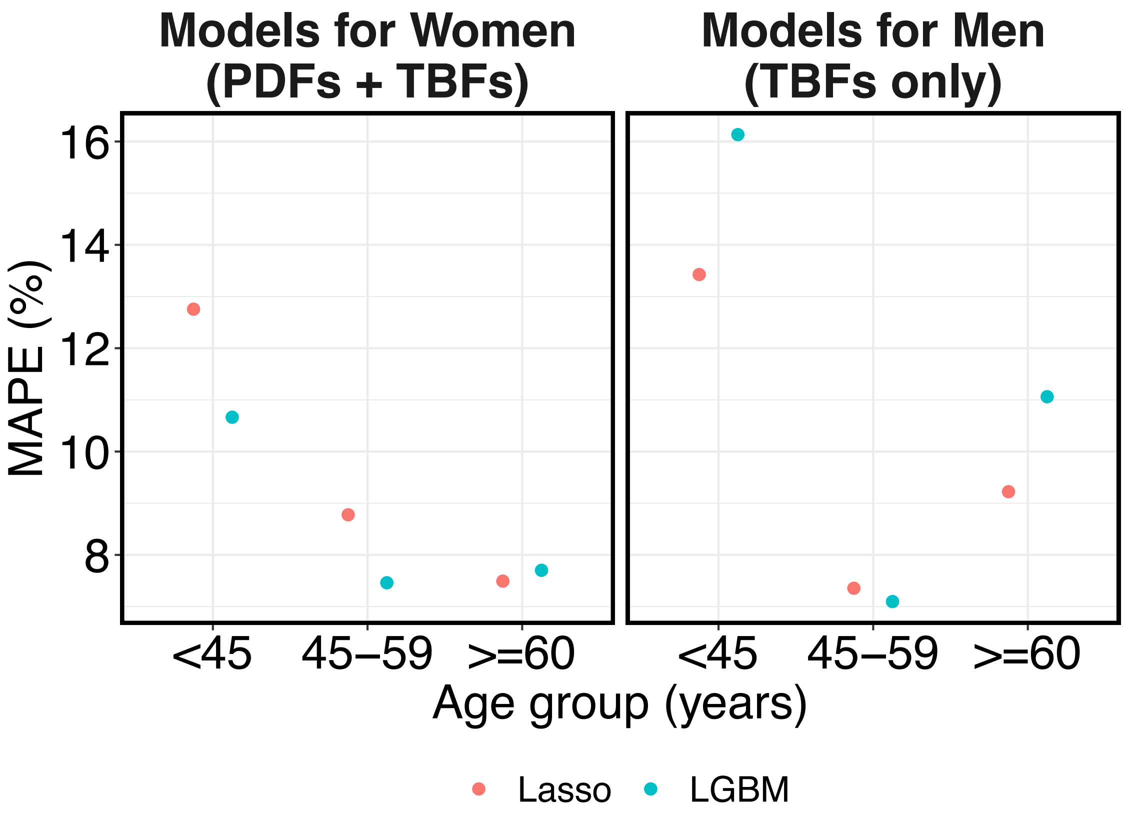

Supplementary Figure S4. Age-stratified MAPE of sex-specific LSFG-based age-prediction models. Mean absolute percentage error (MAPE) of lasso and LightGBM models across three age groups (<45, 45–59, and ≥60 years) is shown. For each sex, the models use the feature sets that yielded the best overall performance in the main analysis: the left panel shows the model for women using predefined LSFG parameters plus tsfresh-derived features, and the right panel shows the model for men using tsfresh-derived features only. Points indicate MAPE values for each age group. Lasso and LightGBM exhibited similar age-related patterns; however, the model for men showed slightly lower MAPE in the 45–59-year age group, consistent with the higher proportion of men in this age range.

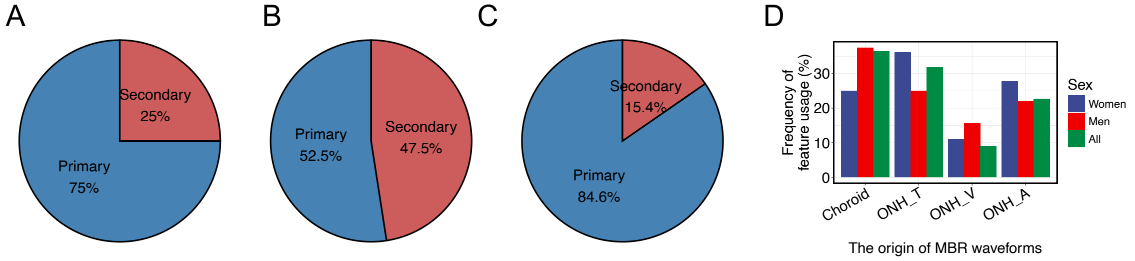

Supplementary Figure S5. Characteristics of features selected in the final models. **(A–C)** Pie charts show the proportions of features derived from primary and secondary waveforms in the final models for women **(A)**, men **(B)**, and all participants **(C)**. **(D)** Bar chart showing the proportions of features derived from different MBR waveform sources (choroid, ONH_T, ONH_V, and ONH_A) used in each of the three models.

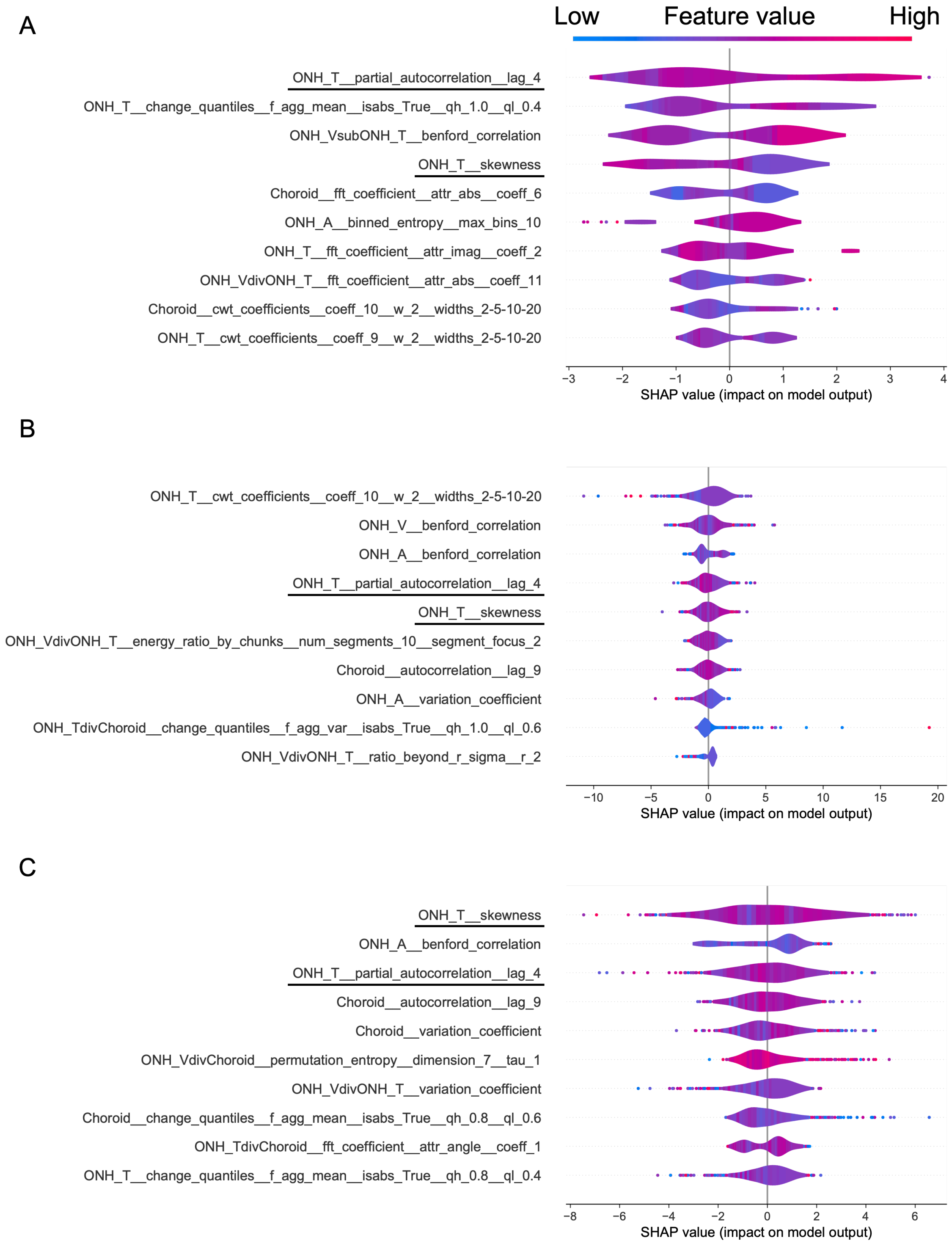

Supplementary Figure S6. The top 10 features, ranked by mean absolute SHAP values, are shown for the best-performing models for women **(A)**, men **(B)**, and all participants **(C)**. Underlined features indicate those shared across all three models.

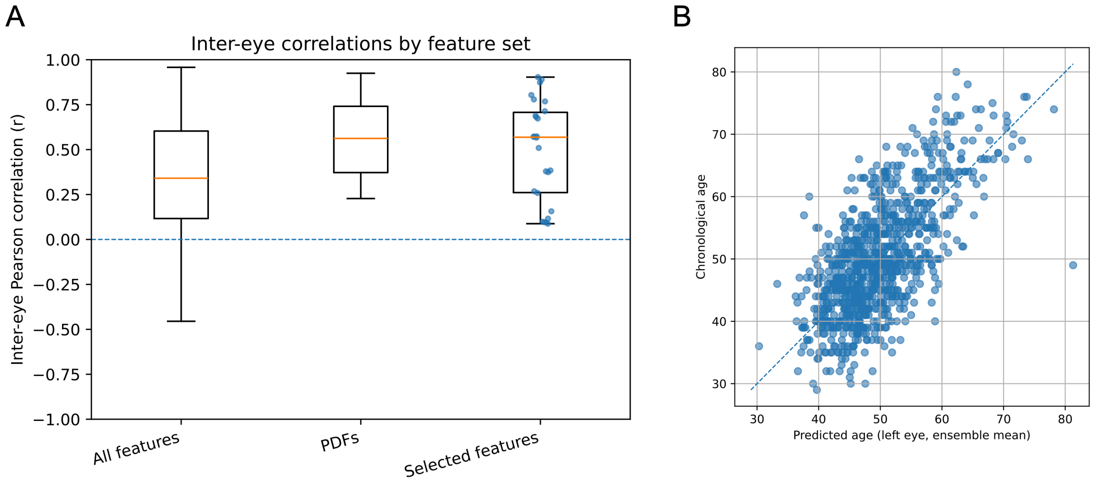

Supplementary Figure S7. Inter-eye consistency of LSFG-derived features and age-prediction performance in left eyes. **(A)** Distributions of Pearson correlation coefficients between right and left eyes for LSFG-derived features in participants with bilateral measurements (n = 903). Left: all features after feature selection (18 predefined parameters and 3,253 tsfresh-derived time-series features), showing a broad distribution that includes both highly symmetric and more eye-specific components. Middle: predefined LSFG parameters alone, showing a relatively eye-specific pattern. Right: subset of tsfresh-derived features that were selected in the best model for all participants, demonstrating a shift toward higher inter-eye correlations compared with the full feature set, indicating that the modeling procedure preferentially relies on features that are more stable between eyes. **(B)** Age-prediction performance of the right-eye–trained model for all participants, when applied to left-eye LSFG data in the bilateral subset (n = 903). The scatter plot shows ensemble-predicted age versus chronological age; the solid line indicates the line of identity. The ensemble achieved a mean absolute error (MAE) of 5.51 years and a mean absolute percentage error (MAPE) of 11.35%, only modestly worse than the corresponding performance for the right eye, supporting the robustness of using right-eye data for model development.

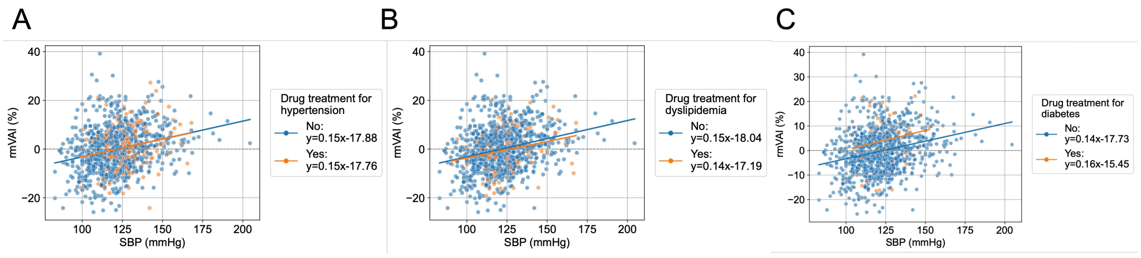

Supplementary Figure S8. Relationship between the relative microvascular ageing index (rmVAI) and systolic blood pressure (SBP), stratified by medication status. The figure illustrates how the association between rmVAI and SBP differs when participants are stratified based on their medication use for common cardiometabolic conditions.

(A-C) Scatter plots showing the relationship between SBP and rmVAI, stratified by medication status. Individuals on medication are shown in orange, and those not on medication are shown in blue. Separate regression lines are fitted for each group. **(A)** Hypertension medication. **(B)** Dyslipidemia medication. **(C)** Diabetes medication.

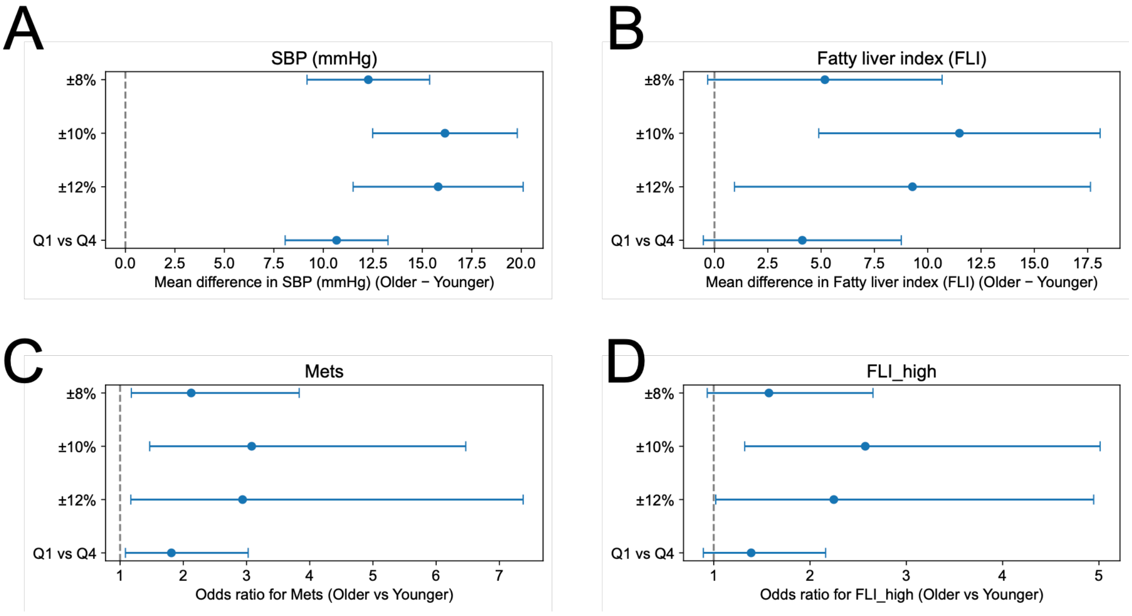

Supplementary Figure S9. Associations between rmVAI-defined microvascular aging status and cardiometabolic risk across alternative thresholds. Forest plots summarizing the associations between the residual microvascular aging index (rmVAI) and key cardiometabolic outcomes under alternative definitions of "model-predicted younger" and "model-predicted older" status. Participants were dichotomized using three absolute rmVAI thresholds (±8%, ±10%, and ±12%) and by quartile-based stratification (Q1 vs Q4). For each definition, "younger" was defined as rmVAI below the lower threshold (or in the lowest quartile), and "older" as rmVAI above the upper threshold (or in the highest quartile), with intermediate values excluded. **(A)** Systolic blood pressure (SBP). Points represent mean differences in SBP (older − younger), and horizontal bars indicate 95% confidence intervals (CIs); the vertical dashed line denotes no difference (0). **(B)** Fatty liver index (FLI). Points represent mean differences in FLI (older − younger) with 95% CIs; the vertical dashed line denotes no difference (0). **(C)** High fatty liver index (FLI_high). Points represent odds ratios (ORs) for high FLI (older vs younger) with 95% CIs on a logarithmic scale; the vertical dashed line denotes an odds ratio of 1.0. **(D)** Metabolic syndrome (MetS). Points represent odds ratios for MetS (older vs younger) with 95% CIs on a logarithmic scale; the vertical dashed line denotes an odds ratio of 1.0.

Across all threshold definitions, the direction and magnitude of the effect estimates were broadly consistent, supporting the robustness of the main findings based on the ±10% rmVAI cut-off.

Supplementary Table S1. Pearson correlation coefficient between chronological and predicted age in each model. Models showing the best performance for women, men, and all participants are shown in bold (see also **Table 1**).

Participant group	Feature set	Models	
		Lasso	LGBM
**Women**	PDFs	0.691	0.616
	TBFs	0.802	0.738
	Both	0.796	**0.838**
**Men**	PDFs	0.681	0.641
	TBFs	**0.783**	0.740
	Both	0.767	0.743
**All**	PDFs	0.670	0.631
	TBFs	0.744	0.731
	Both	**0.745**	0.731

Supplementary Table S2. Number of features used in the ML models. Values in parentheses indicate the number of predefined features (PDFs). Models showing the best performance for women, men, and all participants are shown in bold (see also Table 1).

**Participant group**	**Feature set**	**Models**	
		**Lasso**	**LGBM**
**Women**	PDFs	11	10
	TBFs	14	192
	Both	16 (1)	**48 (3)**
**Men**	PDFs	15	8
	TBFs	**61**	746
	Both	39 (2)	614 (6)
**All**	PDFs	15	10
	TBFs	**26**	228
	Both	27 (1)	777 (9)

Supplementary Table S3. Pearson correlation coefficients (PCCs) between rmVAI and clinical parameters in each sex-group. q-values are Benjamini–Hochberg FDR–adjusted p-values for multiple testing across all clinical variables. Statistical significance is indicated as follows: *q < 0.05.

	**All**			**Women**			**Men**		
**Variable**	**Correlation**	**p-value**	**q-value**	**Correlation**	**p-value**	**q-value**	**Correlation**	**p-value**	**q-value**
**SBP (mmHg)**	0.26*	1.2E-16	3.1E-15	0.30*	7.4E-06	1.9E-04	0.25*	1.1E-12	2.9E-11
**DBP (mmHg)**	0.15*	2.3E-06	2.9E-05	0.20	4.1E-03	5.1E-02	0.14*	6.4E-05	6.4E-04
**BMI (kg/m^2)**	0.04	2.5E-01	5.3E-01	0.02	7.4E-01	7.7E-01	0.04	2.8E-01	5.0E-01
**Waist circumference (cm)**	0.03	3.0E-01	5.7E-01	0.04	5.8E-01	7.2E-01	0.03	4.0E-01	6.2E-01
**FPG (mg/dL)**	0.11*	4.2E-04	2.6E-03	-0.06	3.9E-01	5.4E-01	0.14*	1.2E-04	7.2E-04
**HbA1c (%)**	0.12*	8.7E-04	4.4E-03	0.03	6.9E-01	7.7E-01	0.13*	9.0E-04	4.5E-03
**TG (mg/dL)**	0.09*	5.9E-03	1.6E-02	0.16	2.4E-02	1.2E-01	0.08	1.9E-02	5.3E-02
**HDL (mg/dL)**	-0.02	5.5E-01	7.6E-01	-0.02	7.2E-01	7.7E-01	-0.02	6.7E-01	8.0E-01
**LDL (mg/dL)**	0.01	8.6E-01	9.6E-01	0.16	1.9E-02	1.2E-01	-0.04	2.7E-01	5.0E-01
**γGTP (Unit/L)**	0.10*	2.2E-03	6.9E-03	0.05	4.9E-01	6.4E-01	0.11*	2.9E-03	1.2E-02
**WBC (10^3/mm^3)**	0.10*	1.7E-03	5.9E-03	0.11	1.2E-01	3.5E-01	0.10*	6.0E-03	1.9E-02
**CRP (mg/dL)**	0.05	2.1E-01	4.8E-01	-0.10	1.9E-01	3.5E-01	0.09	2.1E-02	5.3E-02
**Heart rate (bpm)**	-0.02	4.8E-01	7.1E-01	-0.10	1.7E-01	3.5E-01	-0.00	8.9E-01	9.5E-01
**Height (cm)**	-0.06	5.7E-02	1.4E-01	-0.13	5.4E-02	2.3E-01	-0.07	3.5E-02	7.9E-02
**Weight (kg)**	-0.00	9.6E-01	9.6E-01	-0.03	6.8E-01	7.7E-01	-0.00	9.7E-01	9.7E-01
**RBC (10^3/mm^3)**	-0.01	8.3E-01	9.6E-01	0.08	2.2E-01	3.7E-01	-0.03	3.4E-01	5.7E-01
**Plt (10^3/mm^3)**	0.03	3.2E-01	5.7E-01	-0.01	9.4E-01	9.4E-01	0.04	2.6E-01	5.0E-01
**Hb (g/dL)**	0.01	8.1E-01	9.6E-01	0.07	3.1E-01	4.8E-01	-0.02	6.5E-01	8.0E-01
**Ht (%)**	0.00	9.5E-01	9.6E-01	0.06	3.8E-01	5.4E-01	-0.02	5.7E-01	8.0E-01
**Alb (g/dL)**	0.02	6.1E-01	8.1E-01	0.10	1.6E-01	3.5E-01	-0.00	9.1E-01	9.5E-01
**AST (Unit/L)**	-0.00	9.3E-01	9.6E-01	0.09	1.9E-01	3.5E-01	-0.02	6.5E-01	8.0E-01
**ALT (Unit/L)**	0.02	4.6E-01	7.1E-01	0.10	1.6E-01	3.5E-01	0.01	7.5E-01	8.5E-01
**eGFR (mL/min/1.73m²)**	0.13*	2.0E-05	1.6E-04	0.12	8.6E-02	3.1E-01	0.14*	7.7E-05	6.4E-04
**AMY (Unit/L)**	-0.11*	1.1E-03	4.8E-03	-0.11	1.2E-01	3.5E-01	-0.11*	5.0E-03	1.8E-02
**Total Cho (mg/dL)**	0.03	4.1E-01	6.8E-01	0.20	8.9E-03	7.4E-02	-0.02	5.5E-01	8.0E-01

Supplementary Table S4. Detailed summary statistics and effect sizes for clinical parameters shown in Table 4. Summary statistics for all analyzed clinical parameters comparing “model-predicted younger” (rmVAI < −10%) and “model-predicted older” (rmVAI > +10%) groups are presented. For each parameter, group-specific means, standard deviations, and sample sizes (n) are shown, together with mean differences (older − younger) and their 95% confidence intervals (CIs). p-values are based on Mann–Whitney U tests. q-values are Benjamini–Hochberg FDR–adjusted p-values computed jointly across all parameters in this table.

	model-predicted younger			model-predicted older			mean differences (older − younger)				
Variable	Mean	Std	n	Mean	Std	n	Diff	CI95_lower	CI95_upper	p-value	q-value
Age	48.9	8.0	124	49.3	10.6	130	0.4	-1.9	2.7	9.1E-01	9.1E-01
rmVAI (%)	-14.6	3.9	124	15.6	5.0	130	30.2	29.1	31.3	3.8E-43	1.0E-41
SBP (mmHg)	113.8	12.9	123	129.9	16.4	130	16.1	12.5	19.8	7.8E-15	1.0E-13
DBP (mmHg)	71.6	9.9	123	78.8	11.5	130	7.2	4.5	9.8	1.1E-06	9.9E-06
BMI (kg/m^2)	23.2	3.5	123	24.1	3.7	130	0.9	0.0	1.8	6.3E-02	1.3E-01
Waist circumference (cm)	82.8	10.1	123	85.6	10.0	130	2.8	0.3	5.3	1.8E-02	4.6E-02
FPG (mg/dL)	100.0	15.1	122	106.8	21.3	130	6.8	2.2	11.3	9.6E-03	2.8E-02
HbA1c (%)	5.6	0.5	101	5.9	0.9	97	0.2	0.0	0.4	1.4E-01	2.2E-01
TG (mg/dL)	111.5	84.1	123	149.3	154.3	130	37.7	7.2	68.3	2.6E-03	1.3E-02
HDL (mg/dL)	65.5	17.1	123	62.2	17.3	130	-3.3	-7.5	1.0	8.4E-02	1.5E-01
LDL (mg/dL)	125.5	29.3	123	130.5	31.9	130	5.0	-2.6	12.6	2.2E-01	3.0E-01
γGTP (Unit/L)	36.8	42.1	123	54.8	72.8	130	18.1	3.4	32.7	2.3E-03	1.3E-02
WBC ($10^3/mm^3$)	5.6	1.4	123	6.2	1.6	130	0.6	0.2	1.0	3.3E-03	1.3E-02
CRP (mg/dL)	0.1	0.4	99	0.2	0.6	98	0.0	-0.1	0.2	1.5E-01	2.3E-01
Heart rate (bpm)	70.5	10.9	124	69.3	9.5	130	-1.2	-3.7	1.3	5.1E-01	5.7E-01
Height (cm)	67.1	12.6	123	68.6	12.5	130	1.5	-1.6	4.6	2.8E-01	3.6E-01
Weight (kg)	4.7	0.4	123	4.8	0.5	130	0.0	-0.1	0.1	7.5E-01	8.1E-01
RBC ($10^3/mm^3$)	249.7	53.3	123	259.5	54.3	130	9.9	-3.5	23.2	7.7E-02	1.4E-01
Plt ($10^3/mm^3$)	14.3	1.4	123	14.5	1.3	130	0.2	-0.2	0.5	2.2E-01	3.0E-01
Hb (g/dL)	43.3	3.7	123	43.5	3.5	130	0.3	-0.6	1.2	4.9E-01	5.7E-01
Ht (%)	4.6	0.3	112	4.6	0.3	126	0.0	-0.1	0.1	7.8E-01	8.1E-01
Alb (g/dL)	25.6	21.2	123	25.4	11.4	130	-0.2	-4.5	4.0	6.1E-02	1.3E-01
AST (Unit/L)	26.8	24.5	123	29.0	19.7	130	2.2	-3.3	7.7	3.0E-02	7.0E-02

ALT (Unit/L)	75.6	12.9	123	81.8	17.0	130	6.2	2.5	10.0	3.4E-03	1.3E-02
eGFR (mL/min/1.73m²)	74.9	20.7	104	69.2	25.2	112	-5.7	-11.9	0.4	9.0E-03	2.8E-02
AMY (Unit/L)	202.7	35.1	83	209.1	38.6	103	6.4	-4.3	17.1	3.9E-01	4.9E-01
Total Cho (mg/dL)	48.9	8.0	124	49.3	10.6	130	0.4	-1.9	2.7	9.1E-01	9.1E-01
